# Risk factors of low bone mass in young patients with transfusion-dependent beta-thalassemia

**DOI:** 10.3389/fendo.2025.1599437

**Published:** 2025-07-02

**Authors:** Wei Zhang, Rongrong Liu, Siping He, Jihua Huang, Liting Wu, Cuifeng Huang, Yuzhen Liang, Yongrong Lai

**Affiliations:** ^1^ Department of Endocrinology, The Second Affiliated Hospital of Guangxi Medical University, Nanning, China; ^2^ Guangxi Medical University, Nanning, China; ^3^ Department of Hematology, The First Affiliated Hospital of Guangxi Medical University, Nanning, China; ^4^ National Health Commission (NHC) Key Laboratory of Thalassemia Medicine, Nanning, China; ^5^ Guangxi Key Laboratory of Thalassemia Research, Nanning, China

**Keywords:** transfusion-dependent beta-thalassemia, low bone mass, pediatric and adolescents, bone mineral density, height-adjusted

## Abstract

**Objective:**

To determine the prevalence of low bone mass and associated risk factors among children and adolescents suffering from transfusion-dependent beta-thalassemia (TDT).

**Methods:**

In this study, a total of 389 children and adolescents with TDT (236 males and 153 females), treated between January 2015 and December 2024 in the Department of Hematology at the First Affiliated Hospital of Guangxi Medical University, were selected. Subjects were categorized into those with normal bone mass and those with low bone mass based on bone mineral density assessments. Comparative analyses of various indicators between these two groups were performed.

**Results:**

The overall prevalence of low bone mass in TDT patients aged 2–19 years without height-adjusted bone mineral density(BMD)correction was 31.6%, with a prevalence of 33.4% in the 5-19-year subgroup. Multivariate analysis identified age (OR = 1.149, 95% CI 1.052–1.256, P < 0.05), IGF-1 levels < -2 SD (OR = 1.832, 95% CI 1.095–3.067, P < 0.05), and hypogonadism (OR = 2.990, 95% CI 1.087–8.229, P < 0.05) as independent risk factors for low bone mass. After applying height-adjusted BMD correction to the 5-19-year subgroup, the prevalence of low bone mass decreased to 15.8%. In this subgroup, multivariate analysis revealed age (OR = 1.137, 95% CI: 1.034–1.251, P < 0.05), normal BMI (OR = 0.383, 95% CI: 0.158–0.976, P < 0.05), and albumin (ALB) levels (OR = 0.866, 95% CI: 0.783–0.953, P < 0.05) as independent predictors of low bone mass.

**Conclusion:**

This study reveals a high prevalence of low bone mass in children and adolescents with TDT. Without height-adjusted BMD correction, the overall prevalence was 31.6% (33.4% in the 5-19-year subgroup), which significantly decreased to 15.8% in the 5-19-year subgroup after height-adjusted correction, highlighting that traditional BMD assessments may overestimate risk due to unaccounted short stature. Multivariate analysis demonstrated that advancing age consistently remained an independent risk factor (pre-correction OR=1.149; post-correction OR=1.137). The corrected model further identified normal BMI (OR=0.383) and ALB (OR=0.866) as protective factors, while IGF-1 levels < -2 SD (OR=1.832) and hypogonadism (OR=2.990) emerged as significant risks in the uncorrected model. Clinical management should prioritize height-adjusted BMD evaluation and integrated interventions targeting growth hormone axis function, gonadal status, and nutritional indicators to optimize bone health in TDT patients.

## 1 Introduction

Transfusion-Dependent beta -Thalassemia (TDT) is a serious hereditary hemolytic disease caused by defects in the beta-globin genes. It requires long-term blood transfusions and regular iron-chelation therapy to sustain life and is one of the global social public health concerns highlighted by the World Health Organization (WHO) (1). With advancements in standardized transfusion protocols, iron chelation therapy, and hematopoietic stem cell transplantation, the life expectancy of TDT patients has significantly improved. However, this progress has been accompanied by an increased incidence of skeletal complications, particularly osteoporosis and low bone mass (LBM), which have emerged as critical clinical concerns (2–4). Research indicates that 10%-50% of pediatric and adolescent TDT patients develop osteoporosis—significantly exceeding rates in healthy peers—with 20%-40% suffering pathological fractures, severely compromising quality of life and long-term outcomes (5–8). LBM, a precursor to osteoporosis, is especially prevalent in TDT children, characterized by bone mineral density (BMD) below age- and sex-matched norms without meeting osteoporosis diagnostic criteria (9). Adolescence represents a crucial window for bone mass accrual, and failure to intervene early in LBM may lead to progressive bone loss, eventually resulting in osteoporosis and pathological fractures (10).

In our study, we used dual-energy X-ray absorptiometry (DXA) to determine BMD in the lumbar region (L1-L4) for assessing bone mass in TDT children and adolescents. This method was chosen due to the lumbar spine’s high trabecular bone content, optimal for detecting bone mass changes. In pediatric bone density assessment, DXA surpasses QCT (quantitative computed tomography) with its minimal radiation exposure, high standardization, operational simplicity, and cost-effectiveness (9).

The pathogenesis of LBM in TDT children involves multifaceted mechanisms, including genetic predisposition, age and sex factors, anemia severity, disease progression, extramedullary hematopoiesis, iron overload, chelating therapy types/side effects, endocrine dysfunction, growth retardation, hepatic/renal impairment, calcium-phosphorus imbalance, vitamin D deficiency, malnutrition, and physical inactivity (8, 11–14). Current screening and management strategies for LBM in TDT patients remain inadequate, particularly lacking systematic interventions during adolescence—the critical period for bone mass accumulation. Existing studies show ongoing controversies regarding key determinants of LBM, underscoring the urgent need for new research to identify critical risk factors for LBM in TDT children and adolescents. Such investigations could provide theoretical foundations for clinical interventions during this reversible phase, ultimately reducing long-term skeletal complications.

Thalassemia is a prevalent medical condition in Guangxi, China. With a carrier rate of 6.66% for beta-thalassemia genes among the population of Guangxi, it ranks as the province with the highest prevalence in China, thus designating it as a high-incidence region for the disease (15). Unfortunately, comprehensive research on bone mass reduction among Chinese children and adolescents with TDT is currently sparse. Situated within the First Affiliated Hospital of Guangxi Medical University, the Department of Hematology is recognized as a leading national center for hematopoietic stem cell transplantation in the treatment of thalassemia in China. The department has been dedicated to performing hematopoietic stem cell transplantation procedures for TDT patients. This study seeks to investigate BMD and associated influencing factors in pediatric and adolescent patients with TDT scheduled for hematopoietic stem cell transplantation at the Department. The objective is to elucidate the incidence of low bone mass among Chinese children and adolescents afflicted with TDT, pinpoint the predictors of diminished bone density, and assist in the timely recognition of individuals at elevated risk for osteoporosis.

## 2 Methods

### 2.1 Study design

This was a single-center, cross-sectional, retrospective study that used data obtained from the Department of Hematology at the First Affiliated Hospital of Guangxi Medical University. The study has been reviewed and received approval from the Medical Ethics Committee of the First Affiliated Hospital of Guangxi Medical University, under approval number: 2024-S1000-01.

### 2.2 Study population

We selected 389 pediatric and adolescent patients diagnosed with TDT who underwent BMD assessments within the Department of Hematology at the First Affiliated Hospital of Guangxi Medical University between January 2015 and December 2024; all were candidates for hematopoietic stem cell transplantation. Participants were categorized into two cohorts based on BMD assessments: the normal bone mass cohort and the low bone mass cohort. Inclusion criteria included patients diagnosed with Thalassemia major, either through hemoglobin electrophoresis, DNA analysis, or clinical evidence of transfusion dependence, aligning with diagnostic standards set by established guidelines (as documented in discharge or initial outpatient records) and who had undergone a BMD test; patients were less than 20 years of age. Exclusion Criteria: Patients with other known diseases that affect bone quantity; patients on long-term glucocorticoid therapy or other medications that impact bone metabolism; and those with incomplete personal and medical records.

Each patient underwent dual-energy X-ray absorptiometry (DXA; Hologic Horizon-A, Hologic Inc. USA), focusing on the lumbar spine (L1-L4) for bone mineral density assessments. The mean bone mineral density values and related Z-scores were meticulously recorded and subsequently diagnosed. The primary clinical data collected include: Demographic information: age, gender, height, weight, and history of splenectomy. Laboratory parameters included hemoglobin (Hb), albumin (ALB), alkaline phosphatase (ALP), creatinine (Cr), total cholesterol (TC), triglycerides (TG), low-density lipoprotein cholesterol (LDLC), high-density lipoprotein cholesterol (HDLC), calcium (Ca), phosphorus (P), parathyroid hormone (PTH), 25-hydroxyvitamin D [25(OH)D], fasting plasma glucose (FPG), fasting insulin (FINS), serum ferritin (SF), insulin-like growth factor-1 (IGF-1), testosterone (T), estradiol (E2), thyroid hormones (free triiodothyronine [FT3], free thyroxine [FT4], thyroid-stimulating hormone [TSH]), osteocalcin (OC), liver iron concentration (LIC), cardiac MRI T2* (a measure of myocardial iron deposition), type of iron chelator, and lumbar spine bone mineral density (BMD) values with corresponding Z-scores. Height and weight were expressed as SD scores and percentiles, while BMI was presented as percentiles (referencing Chinese growth and development reference norms for children and adolescents). Calculation of BMI= weight (Kg)/height squared (m^2).

### 2.3 Definition

Height: Short Stature: height percentile <3%, Normal: height percentile 3-97%, Tall Stature: >97% (16).

Weight: Underweight: weight percentile <3%, Normal: weight percentile 3-97%, Obesity: >97% (16).

BMI: Thinness: BMI percentile <3%, Normal: BMI percentile 3-85%, Overweight: BMI percentile 85-97%, Obesity: BMI percentile >97% (16).

Low BMD: Characterized by a Z-score of -2.0 or lower at the lumbar spine, as assessed via DXA. In contrast, Normal BMD is denoted by a Z-score greater than -2.0 at the same skeletal site (17).

Hypogonadism (18):Females ≥ 13 years old and males ≥ 14 years old exhibit prepubertal and/or per pubertal progression levels of sex steroids(serum testosterone levels in males < 3.5 nmol/L, serum estradiol levels in females < 50 ng/L).

IGF-1 < -2SD: Using the IGF-1 levels of the general Chinese population of the same sex and age as the reference (19).

Hypothyroidism is characterized by elevated TSH levels beyond the upper limit of the reference range and diminished free T4 levels below the lower limit of the reference range.

Subclinical hypothyroidism is defined by elevated TSH levels surpassing the upper limit of the reference range, in conjunction with free T4 levels that fall within the normal range.

Vitamin D deficiency: Serum levels of 25-hydroxyvitamin D (25(OH)D) are below 50 nmol/L.

Iron overload: Serum ferritin levels are categorized as follows: mild (< 1000 ng/ml), moderate (1000–2500 ng/ml), and severe (> 2500 ng/ml).

### 2.4 Statistical analysis

Data analysis was conducted utilizing Excel and SPSS 27.0 (IBM) software. In instances of normally distributed continuous data, results were presented as the mean ± standard deviation, with intergroup comparisons carried out using the independent samples t-test. For continuous data that were not normally distributed, results were depicted as the median (M) along with the interquartile range (P25-P75), and intergroup comparisons were executed using the Mann-Whitney U test. Categorical variables were presented in terms of frequency counts and percentages, and were analyzed using either the chi-square test or Fisher’s exact test. Variables that reached a significance level of P < 0.05 in the univariate analysis were subsequently incorporated into a binary logistic regression model to determine the independent risk factors associated with reduced bone density in the pediatric and adolescent population with TDT. Statistical significance was defined as a P value of less than 0.05.

## 3 Results

### 3.1 Study population

In the group of 389 enrolled patients, ages spanned from 2 to 19 years, with an average age of 8 ± 3years. The cohort comprised 236 males and 153 females. Low bone mass prevalence was 31.6% overall, slightly lower in males at 30.9% compared to females at 32.7%, with no statistically significant discrepancy noted. Among those with low bone mass, ages varied from a minimum of 3 years to a maximum of 19 years. The prevalence of low bone mass was observed to rise with increasing age. Furthermore, 282 patients (72.5%) exhibited severe iron overload, defined by ferritin levels above 2500 ng/ml, with an average serum ferritin concentration of 3760.620 ng/ml (IQR: 2360.320 to 5502.850 ng/ml). The prevalence of LIC (Liver Iron Concentration) is overall 88.7%, while that for CID (Cardiac Iron Deposition, measured by Cardiac MRI T2*) is 22.4%. The prevalence of chelation therapy usage is as follows: DFO 10.0%, DFP 9.8%, DFX 25.7%, and combined iron chelation 41.3%, with 13.1% not using any or with usage being unknown. A history of splenectomy is present in 12.3% of patients. The prevalence of vitamin D deficiency is 39.1% (152 patients) overall, reaching 36.0% (85 of 236 patients) in males and 43.8% (67 of 153 patients) in females. The prevalence of hypothyroidism is 0.5% (2 of 389 patients), with subclinical hypothyroidism affecting 6.9% (27 of 389 patients) of the population. IGF-1<-2SD is identified in 50.1% (195 of 389 patients) of individuals, with 54.7% (129 of 236 patients) prevalence in males and 43.1% (66 0f 153 patients) in females. Among patients over the age of 13, hypogonadism is prevalent in 70.6% (36 of 51 patients), occurring in 74.2% (23 of 31 patients) of males and 65.0% (13 of 20 patients) of females. Upon comparative analysis of the two groups, the low bone mass group was observed to have higher mean age, lower SDs values for height and weight, increased proportions of short stature and low body weight, elevated TG levels, higher prevalence of hypogonadism and IGF-1<-2SD, alongside significantly reduced BMD and Z-scores at the lumbar spine. No statistically significant differences were observed in other parameters. For detailed results, refer to **Table 1**.

**Table 1:** Baseline characteristics.

Variable	Total (n=389)	Normal BMD (n=266)	Low BMD (n=123)	P
Age [IQR, years]	8.00[5.00,11.00]	7.00[5.00,10.00]	10.00[8.00,13.00]	<0.001
SF [IQR, ng/ml]	3760.62[2360.32,5502.85]	3675.50[2356.74,5179.27]	4053.00[2402.51,6541.92]	0.177
Height SDs [IQR, cm]	-1.00[-2.00,-1.00]	-1.00[-2.00,0.00]	-2.00[-2.00,-1.00]	<0.001
Weight SDs [IQR, Kg]	-1.000[-2.000,0.000]	-1.000[-1.000,0.000]	-2.000[-2.000,-1.000]	<0.001
Hb ( ± SD, g/L)	102.48 ± 16.93	102.44 ± 16.73	102.57 ± 17.36	0.945
ALB ( ± SD, g/L)	42.61 ± 3.19	42.56 ± 2.96	42.74 ± 3.63	0.634
ALP [IQR,U/L]	218.00[176.00,271.00]	215.00[173.00,260.00]	226.00[182.00,308.00]	0.087
Cr [IQR, umol/L]	28.00[24.00,32.00]	28.00[24.00,32.00]	28.00[24.00,32.00]	0.777
TCHO [IQR, mmol/L]	2.94[2.57,3.32]	2.94[2.56,3.30]	2.95[2.58,3.47]	0.627
TG [IQR, mmol/L]	1.08[0.84,1.45]	1.04[0.80,1.39]	1.19[0.91,1.86]	0.002
HDLC [IQR, mmol/L]	0.88[0.74,1.02]	0.89[0.75,1.04]	0.83[0.71,1.01]	0.140
LDLC [IQR, mmol/L]	1.53[1.28,1.85]	1.52[1.24,1.80]	1.56[1.37,1.87]	0.123
Ca [IQR, mmol/L]	2.33[2.26,2.40]	2.33[2.26,2.39]	2.34[2.24,2.41]	0.813
P [IQR, mmol/L]	1.67[1.46,1.81]	1.68[1.51,1.81]	1.66[1.40,1.79]	0.129
FPG [IQR, mmol/L]	4.69[4.35,5.07]	4.69[4.36,5.06]	4.70[4.33,5.16]	0.847
FINS [IQR, pmol/L]	43.06[29.01,63.91]	42.39[29.,58.50]	46.19[27.17,67.63]	0.406
25 (OH)D[IQR, nmol/L]	54.60[44.00,68.40]	56.10[44.50,68.63]	50.50[42.27,67.50]	0.097
PTH [IQR, pg/ml]	28.25[21.28,38.92]	28.42[21.09,38.73]	28.02[21.28,39.90]	0.968
Lumbar Z-score [IQR, SD]	-1.50[-2.20, -0.90]	-1.10[-1.50, -0.60]	-2.50[-3.20, -2.20]	<0.001
BMD [IQR, g/cm2]	0.48[0.43,0.54]	0.49[0.45,0.54]	0.45[0.40,0.54]	<0.001
Lumbar BMD Z score adjusted for height (5–19 year) [IQR, SD] (n=335)	-0.79[-1.40,-0.16]	-0.61[-1.13,0.06]	-2.22[-2.67,-2.02]	<0.001
Gender,n(%)				
Male	236 (60.67)	163 (61.28)	73 (59.35)	0.717
Female	153 (39.33)	103 (38.58)	50 (40.98)	
Hypogonadism, n(%)				
No	353 (90.75)	258 (96.99)	95 (77.24)	<0.001
Yes	36 (9.26)	8 (3.01)	28 (22.76)	
Chelator usage, n(%)				
DFO	39 (10.03)	26 (9.77)	13 (10.57)	0.198
DFP	38 (9.77)	27 (10.15)	11 (8.94)	
DFX	100 (25.71)	75 (28.20)	25 (20.33)	
Combined drugs	161 (41.39)	100 (37.59)	61 (49.59)	
Unused or Unclear	51 (13.11)	38 (14.29)	13 (10.57)	
IGF-1<-2SD, n(%)				
No	194 (49.87)	155 (58.27)	39 (31.71)	<0.001
Yes	195 (50.13)	111 (41.73)	84 (68.29)	
Splenectomy, n(%)				
No	341 (87.66)	237 (89.10)	104 (84.55)	0.205
Yes	48 (12.34)	29 (10.90)	19 (15.45)	
LIC (mg Fe/g dw),n(%)				
No	44 (11.31)	26 (9.77)	18 (14.63)	0.159
Yes	345 (88.69)	240 (90.23)	105 (85.37)	
Cardiac MRI T2* (ms), n(%)				
No	302 (77.64)	206 (77.44)	96 (78.05)	0.894
Yes	87 (22.37)	60 (22.56)	27 (21.95)	
Thyroid Function, n(%)				
Normal	360 (92.55)	246 (92.48)	114 (92.68)	0.944
Abnormal	29 (7.46)	20 (7.52)	9 (7.32)	
OC, n(%)				
Normal	81 (20.82)	54 (20.30)	27 (21.95)	0.709
Abnormal	308 (79.18)	212 (79.70)	96 (78.05)	
Height n(%)				
Short Stature	126 (32.39)	59 (22.18)	67 (54.47)	<0.001
Normal	259 (66.58)	203 (76.32)	56 (45.53)	
Tall Stature	4 (1.03)	4 (1.50)	0 (0.00)	
Weight n(%)				
Underweight	86 (22.11)	33 (12.41)	53 (43.09)	<0.001
Normal	302 (77.64)	232 (87.22)	70 (56.91)	
Obesity	1 (0.26)	1 (0.38)	0 (0.00)	
BMI n(%)				
Thinness	30 (7.71)	17 (6.39)	13 (10.57)	0.045
Normal	334 (85.86)	227 (85.34)	107 (86.99)	
Overweight	19 (4.88)	18 (6.77)	1 (0.81)	
Obesity	6 (1.54)	4 (1.50)	2 (1.63)	

In the subgroup of Lumbar BMD Z score adjusted for height (5–19 years) (n = 335), Normal BMD = 282, Low BMD = 53 annotations: Abnormal thyroid function includes hypothyroidism and subclinical hypothyroidism; Abnormal osteocalcin levels include both elevated and reduced values.

The study population spanned an age range from 2 to 19 years old. Consistent with growth and developmental principles (20), participants were categorized into two age groups: Group 1 included pre-pubertal individuals aged 2–11 years, while Group 2 comprised adolescents aged 12–19 years. Group 1 had an average age of 7 years, while Group 2’s average age was 13. In comparing Group 2 to Group 1, the following characteristics were noted in Group 2: reduced height and weight SDs, an elevated percentage of patients exhibiting low body weight, an increased prevalence of patients with hypogonadism and IGF-1<-2SD, a higher proportion of patients with low bone mass, notably elevated SF concentrations and enhanced CID, a greater incidence of splenectomy, elevated lumbar spine BMD values despite significantly reduced corresponding Z-scores, lower Hb levels, elevated TG and diminished HDLC, increased FPG and INS levels, and diminished 25(OH)D levels (**Table 2**).

**Table 2:** Comparison of patient characteristics between different age groups.

Variable	Total (n=389)	Group1 (n=313)	Group2 (n=76)	P
Gender n (%)				
Male	236 (60.668)	187 (59.744)	49 (64.474)	0.449
Female	153 (39.332)	126 (40.256)	27 (35.526)	
BMI n (%)				
Thinness	30 (7.71)	19 (6.07)	11 (14.47)	0.031
Normal	334 (85.86)	270 (86.26)	64 (84.21)	
Overweight	19 (4.88)	18 (5.75)	1 (1.32)	
Obesity	6 (1.54)	6 (1.92)	0 (0.00)	
Hypogonadism n (%)				
No	353 (90.746)	313 (100.000)	40 (52.632)	<0.001
Yes	36 (9.254)	0 (0.000)	36 (47.368)	
Ironchelators n (%)				
DFO	39 (10.026)	30 (9.585)	9 (11.842)	0.169
DFP	38 (9.769)	30 (9.585)	8 (10.526)	
DFX	100 (25.707)	87 (27.796)	13 (17.105)	
Combined drugs	161 (41.388)	122 (38.978)	39 (51.316)	
Unused or Unclear	51 (13.111)	44 (14.058)	7 (9.211)	
IGF-1<-2SD n (%)				
No	194 (49.871)	185 (59.105)	9 (11.842)	<0.001
Yes	195 (50.129)	128 (40.895)	67 (88.158)	
LBM n (%)				
No	266 (68.380)	236 (75.399)	30 (39.474)	<0.001
Yes	123 (31.620)	77 (24.601)	46 (60.526)	
Splenectomy n (%)				
No	341 (87.661)	287 (91.693)	54 (71.053)	<0.001
Yes	48 (12.339)	26 (8.307)	22 (28.947)	
LIC,n (%)				
No	44 (11.311)	35 (11.182)	9 (11.842)	0.871
Yes	345 (88.689)	278 (88.818)	67 (88.158)	
CardiacMRIT2* n (%)				
No	302 (77.635)	251 (80.192)	51 (67.105)	0.014
Yes	87 (22.365)	62 (19.808)	25 (32.895)	
Thyroidfunction n (%)				
Normal	360 (92.545)	289 (92.332)	71 (93.421)	0.746
Abnormal	29 (7.455)	24 (7.668)	5 (6.579)	
Osteocalcin n (%)				
Normal	81 (20.823)	66 (21.086)	15 (19.737)	0.795
Abnormal	308 (79.177)	247 (78.914)	61 (80.263)	
Age median [IQR]	8.000 [5.000,11.000]	7.000 [5.000,9.000]	13.000 [12.000,15.000]	<0.001
Height SDs median [IQR]	-1.000 [-2.000,-1.000]	-1.000 [-2.000,0.000]	-2.000 [-3.000,-2.000]	<0.001
Weight SDs median [IQR]	-1.000 [-2.000,0.000]	-1.000 [-1.000,0.000]	-2.000 [-3.000,-1.000]	<0.001
Z median [IQR]	-1.500 [-2.200,-0.900]	-1.400 [-1.900,-0.800]	-2.300 [-3.200,-1.300]	<0.001
SF median [IQR]	3760.620 [2360.320,5502.850]	3473.960 [2334.200,5100.690]	5543.400 [3044.110,7993.910]	<0.001
Hb mean ( ± SD)	102.481 ± 16.932	103.377 ± 16.835	98.791 ± 16.831	0.034
Alb mean ( ± SD)	42.614 ± 3.188	42.527 ± 3.193	42.975 ± 3.144	0.273
ALP median [IQR]	218.000 [176.000,271.000]	217.000 [176.000,271.000]	220.000 [176.000,269.000]	0.774
Cr median [IQR]	28.000 [24.000,32.000]	28.000 [24.000,31.000]	30.000 [24.000,36.000]	0.052
TCHO median [IQR]	2.940 [2.570,3.320]	2.970 [2.600,3.350]	2.879 [2.540,3.170]	0.13
TG median [IQR]	1.080 [0.840,1.450]	1.020 [0.790,1.330]	1.390 [1.106,2.090]	<0.001
HDLC median [IQR]	0.880 [0.740,1.020]	0.890 [0.750,1.050]	0.809 [0.700,0.930]	0.002
LDLC median [IQR]	1.530 [1.280,1.850]	1.540 [1.300,1.860]	1.520 [1.270,1.690]	0.472
Ca median [IQR]	2.330 [2.260,2.400]	2.340 [2.270,2.400]	2.310 [2.230,2.390]	0.107
P median [IQR]	1.670 [1.460,1.810]	1.660 [1.480,1.796]	1.690 [1.450,1.880]	0.411
FPG median [IQR]	4.690 [4.350,5.070]	4.660 [4.320,5.020]	4.920 [4.560,5.370]	<0.001
INS median [IQR]	43.060 [29.010,63.905]	40.260 [26.340,56.290]	66.820 [41.540,95.010]	<0.001
25 (OH)D,median [IQR]	54.600 [44.000,68.400]	56.200 [45.200,69.100]	47.900 [37.600,57.100]	<0.001
PTH median [IQR]	28.250 [21.280,38.920]	28.160 [21.460,37.850]	31.090 [19.970,41.010]	0.643
BMDL1L4 median [IQR]	0.480 [0.431,0.539]	0.462 [0.425,0.508]	0.586 [0.535,0.654]	<0.001

### 3.2 Univariate analysis

Given that BMI is a standardized measure calculated from height and weight, we opted to include BMI in the analysis while excluding height and weight to mitigate multicollinearity among independent variables. Univariate analysis revealed statistically significant differences (P<0.05) in Age, ALP, TG, P, the proportion of individuals with Overweight BMI (85-97%), Hypogonadism and IGF-1<-2SD (**Supplementary Material**). The correlation analysis revealed no significant associations among the seven variables(r<0.5). (**Supplementary Material**). In multivariable logistic regression analysis, with low bone mass as the dependent variable, Age, ALP, TG, P, BMI, Hypogonadism and IGF-1<-2SD were included as independent variables to investigate their associations with the outcome.

### 3.3 Multivariate logistic regression analysis

**Figure 1:**
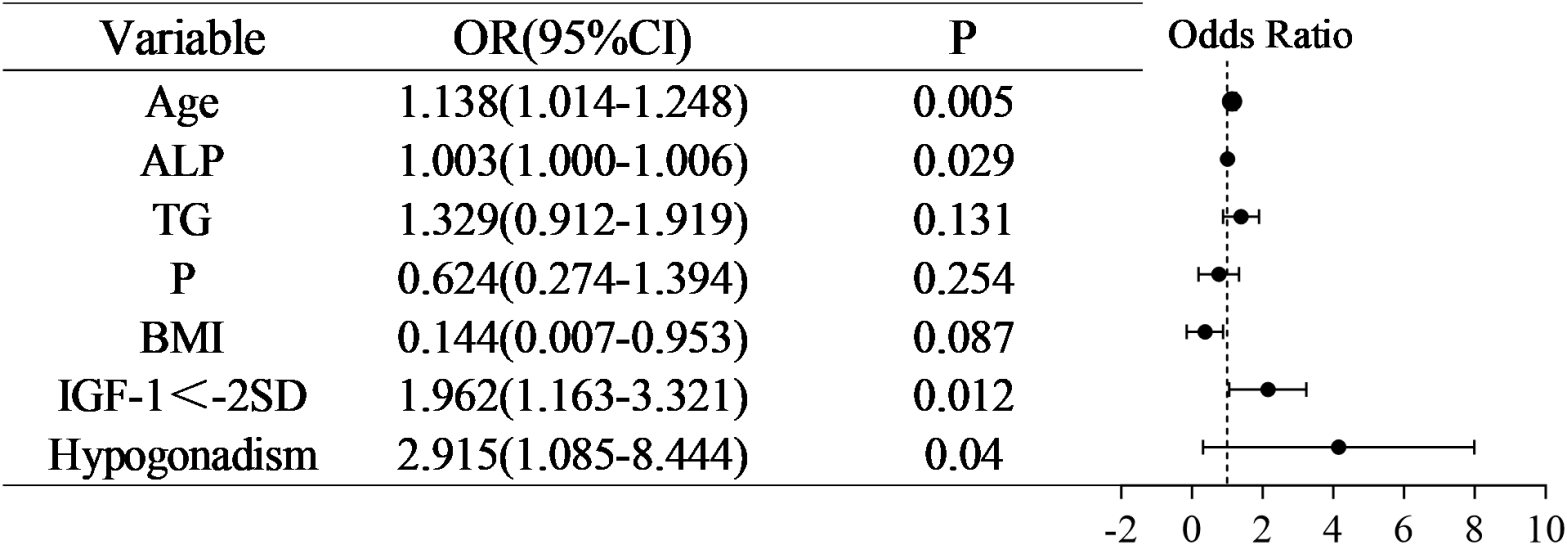
Forest plot for multivariate regression analysis of the entire group.

Following a comprehensive multivariate logistic regression analysis of seven key variables, we identified Age (OR =1.138, 95% CI 1.041-1.248, P < 0.05), IGF-1<-2SD (OR =1.962, 95% CI1.163-3.321, P < 0.05) and Hypogonadism (OR =2.951, 95% CI1.085-8.444, P < 0.05) as independent risk factors for low bone mass in the pediatric and adolescent population with TDT, as outlined in **Figure 1**.

### 3.4 Subgroup analysis

In the 5–19-year patient subgroup, we applied a pediatric bone density calculator (https://zscore.research.chop.edu/calcpedbonedens.php) to perform height-adjusted BMD correction. After correction, the prevalence of low bone mass in this age group decreased from 33.4% to 15.8%. In this subgroup, univariate and multivariate logistic regression analyses revealed that Age, normal BMI, and ALB levels were independent predictors of low bone mass. Specifically, Age was identified as a risk factor (OR = 1.137, 95% CI: 1.034–1.251, P < 0.05), while normal BMI (OR = 0.383, 95% CI: 0.158–0.976, P < 0.05) and ALB levels (OR = 0.866, 95% CI: 0.783–0.953, P < 0.05) were protective factors (**Table 3**).

**Table 3:** Multivariate logistic regression analysis in the 5–19 years subgroup.

Variable	P	OR	95%CI
ALB	0.004	0.866	0.783 - 0.953
ALP	0.071	1.003	1.000 - 1.006
P	0.117	0.443	0.158 - 1.212
Age	0.008	1.137	1.034 - 1.251
BMI	0.037	0.383	0.158 - 0.976

TDT, transfusion-dependent beta-thalassemia; BMD, Bone mineral density; DXA, dual-energy X-ray absorptiometry; Hb, Hemoglobin; ALB, Albumin; ALP, Serum Alkaline Phosphatase; Cr, Creatinine; TC, Total Cholesterol; TG, Triglycerides; LDLC, Low-Density Lipoprotein Cholesterol; HDLC, High-Density Lipoprotein Cholesterol; Ca, Serum Calcium; P, Serum Phosphorus; PTH, Parathyroid Hormone; [25(OH)D],25-Hydroxy Vitamin D; FPG, Fasting Plasma Glucose; FINS, Fasting Insulin Levels; SF, Serum Ferritin; IGF-1,Insulin-like Growth Factor 1; T, Testosterone; E2,Estradiol; OC, Osteocalcin; LIC, Liver Iron Concentration; CID, Cardiac Iron Deposition (measured by Cardiac MRI T2*); BMI, Body Mass Index; DFO, Deferoxamine; DFP, Deferiprone; DFX, Deferasirox; GH, Growth hormone; IGF-1,Insulin-like growth factor 1; IGF-BP3, Insulin-like Growth Factor Binding Protein 3.

## 4 Discussion

Thalassemia-related bone disease is a prevalent complication among patients with Thalassemia major. Distinct from prior research that primarily considered adult patient populations with TDT, our study shifts its focus to pediatric and adolescent patients affected by TDT, investigating the prevalence and determinants of low bone mass within this youthful demographic.

Low bone mass, a condition with reduced bone mineral density but not classified as osteoporosis, has pivotal clinical impacts. It increases fracture risk due to weakened bones, signals impending osteoporosis, necessitating intervention to prevent further bone loss, and offers a window to assess skeletal health, enabling early identification and management of bone issues. For LBM patients, personalized treatment can mitigate bone mass loss. Essentially, the core clinical significance of LBM lies in its value as an “intervention window”—early detection and intervention can delay or halt progression to osteoporosis, reduce fracture risk, and enhance patients’ long-term quality of life (10).

Our study demonstrates that the overall prevalence of low bone mass in children and adolescents with TDT is 31.6%, with the prevalence in the 5–19-year subgroup decreasing to 15.8% after height-adjusted BMD correction. Previous studies reported a prevalence of low bone mass in TDT patients ranging from 40% to 50% (8, 21). he lower prevalence observed in our cohort may be attributed to potential explanations:1) the relatively younger age of our patient cohort, 2)traditional BMD assessments may overestimate prevalence due to the lack of height-adjusted correction. However, even after height correction, the prevalence of low bone mass in TDT patients remains higher than that in the general population, indicating that TDT patients are vulnerable to bone disease from childhood. These findings underscore the critical need for implementing early BMD screening and timely therapeutic interventions in this population to mitigate long-term skeletal complications.

In this cross-sectional study of children and adolescents diagnosed with TDT, we identified that without height-adjusted BMD correction, age (OR = 1.138, 95% CI 1.041–1.248, P 0.05), IGF-1<-2SD (OR = 1.962, 95% CI 1.163–3.321, P 0.05), and hypogonadism (OR = 2.951, 95% CI 1.085–8.444, P 0.05) were significant independent risk factors associated with low bone mass.

Our research identified that advancing age serves as a significant independent risk factor for low bone mass in pediatric and adolescent patients with TDT, corroborating the findings from numerous studies (3, 10, 22, 23). In alignment with the developmental trend of human bone density, there exists an accumulation and increase phase prior to the age of 20, characterized by swift skeletal growth and osteoblastic activity that exceeds osteoclastic activity. Consequently, bone mass progressively accumulates (24). Over a quarter of one’s peak bone mass is rapidly accrued during adolescence, and by age 18, an individual has usually reached 90% of their maximum bone mass (25), which is a critical time for the accumulation of peak bone mass. Participants in our study ranged in age from 2 to 19 years, with an average age of 8 ± 3 years, coinciding with the critical period for peak bone mass accumulation. With increasing age, patients exhibited a rising trend in bone density, yet their respective Z-scores declined. This suggests that the patients’ bone density increase was markedly inferior to the normative values for a comparable demographic. This outcome aligns with previously reported studies (21, 23). With advancing age in TDT patients, there is a progressive accumulation of skeletal risks. The burden of accumulated iron significantly exacerbates the impairment of osteoblast function. Persistent anemia results in bone marrow expansion and cortical thinning. Additionally, the cumulative effects of long-term complications associated with transfusions, including endocrine dysfunction, further complicate the clinical picture. Adolescence represents a pivotal period for substantial bone mass accretion. If impaired by the aforementioned factors, it can result in a substantial decrease in peak bone mass and heighten the risk of osteoporosis in later life (23). Hence, prioritizing the detection and prevention measures among high-risk individuals for osteoporosis during the formative years in children and adolescents with TDT is crucial for enhancing peak bone mass achievement in adulthood and mitigating the risk of osteoporosis later in life.

Hypogonadism represents one of the prevalent endocrine disorders observed in individuals affected by TDT, with estimated prevalence varying between 20% and 77.5% (26). In our study, we found a high prevalence of hypogonadism at 70.6%among patients aged 13 and older, with male patients having a prevalence of 74.2% and female patients at 65.0%.Our research demonstrated a significant correlation between hypogonadism and low bone mass, aligning with prior studies conducted on patients with TDT (8, 10, 22, 27). Sex steroid hormones, namely estrogen and testosterone, are crucial for maintaining the equilibrium of bone remodeling processes. Iron accumulation may result in decreased levels of sex hormones, either by directly damaging gonadal cells or by inhibiting the function of the hypothalamic-pituitary-gonadal axis. Consequently, this disruption affects the estrogen/androgen-regulated pathways crucial for bone metabolism (28), resulting in diminished osteoblast function and augmented bone resorption by osteoclasts (29, 30). Prior research has predominantly concentrated on adults, our research establishes a link in children and adolescents, suggesting that gonadal dysfunction could intervene in early skeletal development, severely impacting the attainment of peak bone mass, and substantially elevating the risk of osteoporosis in adulthood (31). Consequently, there is a pressing need to enhance scrutiny over gonadal function and to implement timely hormone replacement therapy for adolescent patients.

In our investigation, the incidence of IGF-1<-2SD reached 50.1%, with males exhibiting a higher incidence at 54.7% compared to females at 43.1%. In 2022, a study by Gong Chunxiu et al. delineated the standard levels and trends of IGF-1 in Chinese children and adolescents, spanning the age range of 1 to 19 years (19):Serum IGF-1 levels increase with age in both genders, displaying a notable surge in boys around 12–13 years of age, peaking around 15 years of age. In girls, a similar surge is observed at 10–11 years of age, with levels peaking between 12 and 13 years of age. In our study, boys aged 15 exhibited the highest IGF-1 levels at 272.00 ng/ml, which is within the range of -1 to -2 SD below the mean for the general population. The average IGF-1 level among these boys was 141.64 ng/ml, which is significantly lower than the average for the normal population, at 411.50 ng/ml. For girls, at age 12, the highest IGF-1 level reached 285.00 ng/ml, situating between -1 and -2 SD from the norm, with an average of 167.89 ng/ml. This average is considerably below the typical average for the normal population, which stands at 442.00 ng/ml. At age 13, girls’ IGF-1 levels peaked at 257.00 ng/ml, which is below -2SD from the mean of the normal population, and the average was 158.03 ng/ml. This average value is substantially below the average for the normal population of 456.00 ng/ml. These findings suggest that children and adolescents diagnosed with TDT exhibit markedly decreased growth hormone levels when compared to their unaffected peers within the same racial, age, and gender groups. Studies have revealed that the GH/IGF-1 axis is pivotal for regulating bone hyperplasia across prepubertal, pubertal, and postpubertal developmental phases. This axis is also essential for modulating peak bone density and bone mass acquisition during pubertal development (8). In individuals with thalassemia, it has been shown that bone mineral density is correlated with the blood levels of IGF-1 and IGFBP-3 (32). GH facilitates chondrocyte proliferation and the mineralization of bone matrix by enhancing liver production of IGF-1. However, iron overload-induced toxicity in the anterior pituitary may result in reduced GH secretion, leading to osteoporosis and demineralization in TDT patients (33). These alterations contribute significantly to the degradation of bone mineralization. Notably, compared to adults, the detrimental effects of markedly decreased growth hormone levels on bone density are more pronounced in children and adolescents.

Iron overload is widely regarded as a primary cause of reduced bone mass in patients with TDT (34), however, several previous studies have failed to demonstrate a substantial correlation between elevated serum ferritin levels and decreased bone mass (3, 21, 34). In this study, we presented the measurement results for LIC and CID. We also compared the serum ferritin levels, LIC, and CID across the two groups. However, no significant correlation was observed between these independent variables and low bone mass. Possible reasons for the observed discrepancies may include: 1) The study population consisted of children and adolescents, whereas long-term effects of iron overload are potentially more evident in adults. 2) The variability in iron chelation therapy, such as differences in drug selection and patient adherence, could confound the relationship between iron burden and bone metabolism. 3) Administration of iron chelators may mitigate some of the direct toxic effects of iron accumulation on bone, underscoring the critical need for uniform iron chelation therapies. Moreover, aligning with Nakavachara P et al.’s findings, this study likewise failed to detect any substantial influence of hemoglobin and vitamin D levels on osteoporosis or reduced bone mineral density (35).

In the 5–19-year patient subgroup, the significant reduction in the prevalence of low bone mass after height-adjusted correction highlights the critical role of stature in BMD assessment for this age group. Univariate and multivariate logistic regression analyses demonstrated that advancing age was an independent risk factor for low bone mass, with increasing age correlating with elevated risk—a finding consistent with the uncorrected model. In the height-adjusted model, normal BMI and higher ALB levels emerged as protective factors. Normal BMI may reflect adequate nutritional status and optimal levels of bone-modulating factors secreted by adipose tissue, while ALB, as a marker of nutritional health, likely supports skeletal integrity through its role in maintaining metabolic homeostasis. Although IGF-1<-2SD and hypogonadism did not retain significance in the height-adjusted model, their high-risk associations in the uncorrected model suggest that growth hormone axis dysfunction and gonadal hormone deficiency remain potential underlying drivers of bone loss in TDT patients. These findings underscore the need to integrate endocrine and nutritional evaluations alongside height-adjusted BMD assessments to comprehensively address multifactorial bone health challenges in this population.

Our study compared two groups divided by developmental stages (Group 1: pre-pubertal, 2–11 years; Group 2: pubertal and adolescent, 12–19 years) and found that children and adolescents with TDT exhibited significant age-related deteriorations, including impaired growth parameters, increased malnutrition, worsening anemia and iron overload, glucose and lipid metabolism disorders, compromised secretion of sex hormones and growth hormone, and reduced vitamin D levels. These findings indicate that pubertal and adolescent patients face more severe challenges, such as growth retardation, hypogonadism, growth hormone deficiency, skeletal abnormalities, iron overload, and metabolic dysregulation. This underscores the necessity for dynamic monitoring and intervention targeting growth, development, and metabolic parameters during adolescence, alongside multisystem comprehensive interventions, to improve long-term outcomes and quality of life.

This study’s clinical significance is underscored by its identification of independent risk factors for low bone mass in TDT patients, spanning from childhood through adolescence, within a large-sample, retrospective framework. This finding furnishes a theoretical underpinning for the management of bone health. However, several inherent limitations are associated with the study. Firstly, due to its cross-sectional design, this study cannot establish causality and necessitates longitudinal studies to confirm the dynamic effects of risk factors. Secondly, the inclusion of cases solely from one center cannot be overlooked; it introduces potential bias and reduces the study’s statistical power. Thirdly, this study failed to account for height-related BMD across the entire patient cohort and did not conduct assessments for vertebral fractures (VFs).

## 5 Conclusion

Our study revealed that the prevalence of low bone mass in TDT children and adolescents decreased after height-adjusted correction, underscoring the critical role of stature adjustment in BMD assessment. Multivariate analysis consistently identified advancing age as an independent risk factor for low bone mass, normal BMI and ALB as protective factors, while IGF-1 levels < -2 SD and hypogonadism demonstrated significant risk associations in the uncorrected model. Based on these findings, we advocate that clinical management of TDT pediatric and adolescent patients should prioritize height-adjusted BMD evaluation and implement comprehensive interventions targeting growth hormone axis function, gonadal status, and nutritional indicators to optimize bone health outcomes.

## Data Availability

The data analyzed in this study is subject to the following licenses/restrictions: The data that support the findings of this study are available from the corresponding author upon reasonable request. Requests to access these datasets should be directed to Yongrong Lai, laiyongrong@263.net.

## References

[1] Red Blood Cell Diseases (Anemia) Group CSoH. Chinese Medical Association. Chinese guideline for diagnosis and treatment of transfusion dependent β-thalassemia (2022). Chin J Hematol. (2022) 43:889–96. doi: 10.3760/cma.j.issn.0253-2727.2022.11.002 PMC9808868 36709178

[2] Shamoon RP Yassin AK Omar N Saeed MD Akram R Othman NN. Magnitude of bone disease in transfusion-dependent and non-transfusion-dependent β-thalassemia patients. Cureus. (2024) 16:e56012. doi: 10.7759/cureus.56012 38606231 PMC11007755

[3] Bordbar M Bozorgi H Saki F Haghpanah S Karimi M Bazrafshan A. Prevalence of endocrine disorders and their associated factors in transfusion-dependent thalassemia patients: a historical cohort study in Southern Iran. J Endocrinol Invest. (2019) 42:1467–76. doi: 10.1007/s40618-019-01072-z 31228105

[4] Taher AT Musallam KM Cappellini MD. β-thalassemias. N Engl J Med. (2021) 384:727–43. doi: 10.1056/NEJMra2021838 33626255

[5] Shamshirsaz AA Bekheirnia MR Kamgar M Pakbaz Z Tabatabaie SM Bouzari N. Bone mineral density in Iranian adolescents and young adults with beta-thalassemia major. Pediatr Hematol Oncol. (2007) 24:469–79. doi: 10.1080/08880010701533702 17786783

[6] Udeze C Ly NF Ingleby FC Fleming SD Conner SC Howard J. Clinical burden and healthcare resource utilization associated with managing transfusion-dependent β-thalassemia in England. Clin Ther. (2025) 47:37–43. doi: 10.1016/j.clinthera.2024.09.024 39488494

[7] Al-Agha AE Bawahab NS Nagadi SA Alghamdi SA Felemban DA Milyani AA. Endocrinopathies complicating transfusion-dependent hemoglobinopathy. Saudi Med J. (2020) 41:138–43. doi: 10.15537/smj.2020.2.24845 PMC7841625 32020146

[8] De Sanctis V Soliman AT Elsedfy H Yassin M Canatan D Kilinc Y. Osteoporosis in thalassemia major: an update and the I-CET 2013 recommendations for surveillance and treatment. Pediatr Endocrinol Rev. (2013) 11:167–80. 24575552

[9] Crabtree NJ Arabi A Bachrach LK Fewtrell M El-Hajj Fuleihan G Kecskemethy HH. Dual-energy X-ray absorptiometry interpretation and reporting in children and adolescents: the revised 2013 ISCD Pediatric Official Positions. J Clin Densitom. (2014) 17:225–42. doi: 10.1016/j.jocd.2014.01.003 24690232

[10] Vogiatzi MG Macklin EA Fung EB Cheung AM Vichinsky E Olivieri N. Bone disease in thalassemia: a frequent and still unresolved problem. J Bone Miner Res. (2009) 24:543–57. doi: 10.1359/jbmr.080505 PMC3276604 18505376

[11] Di Paola A Marrapodi MM Di Martino M Giliberti G Di Feo G Rana D. Bone health impairment in patients with hemoglobinopathies: from biological bases to new possible therapeutic strategies. Int J Mol Sci. (2024) 25(5):2902. doi: 10.3390/ijms25052902 38474150 PMC10932404

[12] De Sanctis V Soliman AT Elsefdy H Soliman N Bedair E Fiscina B. Bone disease in β thalassemia patients: past, present and future perspectives. Metabolism. (2018) 80:66–79. doi: 10.1016/j.metabol.2017.09.012 28987275

[13] Evangelidis P Venou TM Fani B Vlachaki E Gavriilaki E. Endocrinopathies in hemoglobinopathies: what is the role of iron? Int J Mol Sci. (2023) 24(22):16263. doi: 10.3390/ijms242216263 38003451 PMC10671246

[14] Bain B. 2021 Guidelines for the management of transfusion dependent thalassaemia (TDT). Br J Haematology. (2023) 200:532. doi: 10.1111/bjh.18567 38683909

[15] Writing Group for Practice Guidelines for Diagnosis and Treatment of Genetic Diseases MGBoCMA. Clinical practice guidelines for β-thalassemia. Chin J Med Genet. (2020) 37:243–51. doi: 10.3760/cma.j.issn.1003-9406.2020.03.004 32128739

[16] Li H Ji CY Zong XN Zhang YQ. Height and weight standardized growth charts for Chinese children and adolescents aged 0 to 18 years. Chin J Pediatrics. (2009) 47:487–92. 19951507

[17] Rauch F Plotkin H DiMeglio L Engelbert RH Henderson RC Munns C. Fracture prediction and the definition of osteoporosis in children and adolescents: the ISCD 2007 Pediatric Official Positions. J Clin Densitom. (2008) 11:22–8. doi: 10.1016/j.jocd.2007.12.003 18442750

[18] Association PEGaMGoCMD Association AHaMPCoCMD the Subspecialty Group of Endocrinological HaMD the Society of Pediatrics Chinese Medical Association. Expert consensus on diagnosis and treatment of hypogonadotropic hypogonadism in children. Chin J Pediatr. (2023) 61:484–90. doi: 10.3760/cma.j.cn112140-20221208-01034 37312457

[19] Cao B Peng Y Song W Peng X Hu L Liu Z. Pediatric continuous reference intervals of serum insulin-like growth factor 1 levels in a healthy chinese children population - based on PRINCE study. Endocr Pract. (2022) 28:696–702. doi: 10.1016/j.eprac.2022.04.004 35430364

[20] Parent AS Teilmann G Juul A Skakkebaek NE Toppari J Bourguignon JP. The timing of normal puberty and the age limits of sexual precocity: variations around the world, secular trends, and changes after migration. Endocr Rev. (2003) 24:668–93. doi: 10.1210/er.2002-0019 14570750

[21] Mohseni F Mohajeri-Tehrani MR Larijani B Hamidi Z. Relation between BMD and biochemical, transfusion and endocrinological parameters in pediatric thalassemic patients. Arch Osteoporos. (2014) 9:174. doi: 10.1007/s11657-014-0174-3 24652076

[22] Vichinsky EP. The morbidity of bone disease in thalassemia. Ann N Y Acad Sci. (1998) 850:344–8. doi: 10.1111/j.1749-6632.1998.tb10491.x 9668556

[23] Vogiatzi MG Autio KA Mait JE Schneider R Lesser M Giardina PJ. Low bone mineral density in adolescents with beta-thalassemia. Ann N Y Acad Sci. (2005) 1054:462–6. doi: 10.1196/annals.1345.063 16339698

[24] Golden NH Abrams SA. Optimizing bone health in children and adolescents. Pediatrics. (2014) 134:e1229–43. doi: 10.1542/peds.2014-2173 25266429

[25] Stagi S Cavalli L Iurato C Seminara S Brandi ML de Martino M. Bone metabolism in children and adolescents: main characteristics of the determinants of peak bone mass. Clin cases Miner Bone Metab. (2013) 10:172–9. PMC3917578 24554926

[26] Venou TM Barmpageorgopoulou F Peppa M Vlachaki E. Endocrinopathies in beta thalassemia: a narrative review. Hormones (Athens). (2024) 23:205–16. doi: 10.1007/s42000-023-00515-w 38103163

[27] Gaudio A Morabito N Catalano A Rapisarda R Xourafa A Lasco A. Pathogenesis of thalassemia major-associated osteoporosis: A review with insights from clinical experience. J Clin Res Pediatr Endocrinol. (2019) 11:110–7. doi: 10.4274/jcrpe.galenos.2018.2018.0074 PMC6571534 29991466

[28] Zhuannan J Liyang L. Research progress in hypogonadism of patients with β-thalassemia major. Int J Pediatrics. (2019) 46:424–6. doi: 10.3760/cma.j.issn.1673-4408.2019.06.010

[29] Thavonlun S Houngngam N Kingpetch K Numkarunarunrote N Santisitthanon P Buranasupkajorn P. Association of osteoporosis and sarcopenia with fracture risk in transfusion-dependent thalassemia. Sci Rep. (2023) 13:16413. doi: 10.1038/s41598-023-43633-6 37775530 PMC10541420

[30] Ananvutisombat N Tantiworawit A Punnachet T Hantrakun N Piriyakhuntorn P Rattanathammethee T. Prevalence and risk factors predisposing low bone mineral density in patients with thalassemia. Front Endocrinol (Lausanne). (2024) 15:1393865. doi: 10.3389/fendo.2024.1393865 38978629 PMC11228236

[31] Carsote M Vasiliu C Trandafir AI Albu SE Dumitrascu MC Popa A. New entity-thalassemic endocrine disease: major beta-thalassemia and endocrine involvement. Diagnostics (Basel). (2022) 12(8):1921. doi: 10.3390/diagnostics12081921 36010271 PMC9406368

[32] Lasco A Morabito N Gaudio A Crisafulli A Meo A Denuzzo G. Osteoporosis and beta-thalassemia major: role of the IGF-I/IGFBP-III axis. J Endocrinol Invest. (2002) 25:338–44. doi: 10.1007/BF03344015 12030605

[33] Soliman AT De Sanctis V Elalaily R Yassin M. Insulin-like growth factor- I and factors affecting it in thalassemia major. Indian J Endocrinol Metab. (2015) 19:245–51. doi: 10.4103/2230-8210.131750 PMC4319264 25729686

[34] Dede AD Trovas G Chronopoulos E Triantafyllopoulos IK Dontas I Papaioannou N. Thalassemia-associated osteoporosis: a systematic review on treatment and brief overview of the disease. Osteoporos Int. (2016) 27:3409–25. doi: 10.1007/s00198-016-3719-z 27503175

[35] Nakavachara P Weerakulwattana P Pooliam J Viprakasit V. Bone mineral density in primarily preadolescent children with hemoglobin E/β-thalassemia with different severities and transfusion requirements. Pediatr Blood Cancer. (2022) 69:e29789. doi: 10.1002/pbc.29789 35652568

