# Supplementary material for: Risk factors of low bone mass in young patients with transfusion-dependent beta-thalassemia
Source: Front Endocrinol (Lausanne). 2025 Jul 2;16:1599437. doi: 10.3389/fendo.2025.1599437 (PMC12263410; doi:10.3389/fendo.2025.1599437)
Supplement: Supplementary file 1 [file DataSheet1.docx]

Supplementary Material

# Supplementary Tables

Table Univariate logistic regression analysis of influencing factors for low bone mass

| Variable | P | OR | 95%CI |
| --- | --- | --- | --- |
| Age | <0.001 | 1.267 | 1.183-1.357 |
| SF | 0.104 | 1 | 1.000-1.000 |
| Height SDs | <0.001 | 0.566 | 0.45,0.711 |
| Weight SDs | <0.001 | 0.445 | 0.348,0.568 |
| Hb | 0.944 | 1 | 0.988-1.013 |
| Alb | 0.607 | 1.018 | 0.952-1.088 |
| ALP | 0.011 | 1.003 | 1.001-1.005 |
| Cr | 0.753 | 1.005 | 0.976-1.034 |
| TCHO | 0.176 | 1.284 | 0.894-1.844 |
| TG | 0.007 | 1.545 | 1.126-2.120 |
| HDLC | 0.382 | 0.683 | 0.291-1.604 |
| LDLC | 0.303 | 1.160 | 0.874-1.539 |
| Ca | 0.998 | 1.002 | 0.156-6.422 |
| P | 0.042 | 0.477 | 0.234-0.974 |
| FPG | 0.307 | 1.717 | 0.865-1.585 |
| FINS | 0.072 | 1.004 | 1.000-1.008 |
| PTH | 0.350 | 0.994 | 0.983-1.006 |
| 25（OH）D | 0.339 | 0.995 | 0.984-1.006 |
| Gender |  |  |  |
| Male |  |  |  |
| Female | 0.717 | 1.084 | 0.701-1.677 |
| Hypogonadism |  |  |  |
| No |  |  |  |
| Yes | <0.001 | 9.505 | 4.186-21.586 |
| Chelator usage |  |  |  |
| DFO |  |  |  |
| DFP | 0.678 | 0.815 | 0.310-2.143 |
| DFX | 0.324 | 0.667 | 0.298-1.491 |
| Combined drugs | 0.597 | 1.220 | 0.583-2.552 |
| Unused | 0.417 | 0.684 | 0.274-1.711 |
| IGF-1＜-2SD |  |  |  |
| No |  |  |  |
| Yes | <0.001 | 3.008 | 1.915-4.723 |
| Splenectomy |  |  |  |
| No |  |  |  |
| Yes | 0.207 | 1.493 | 0.801-2.783 |
| LIC |  |  |  |
| No |  |  |  |
| Yes | 0.162 | 0.632 | 0.332-1.202 |
| Cardiac MRI T2* |  |  |  |
| No |  |  |  |
| Yes | 0.894 | 0.966 | 0.577-1.616 |
| ThyroidFunction |  |  |  |
| Normal |  |  |  |
| Abnormal | 0.944 | 0.971 | 0.429-2.199 |
| OC |  |  |  |
| Normal |  |  |  |
| Abnormal | 0.709 | 0.906 | 0.538-1.525 |
| BMI n (%) |  |  |  |
| Underweight |  |  |  |
| Normal | 0.211 | 0.616 | 0.289-1.315 |
| Overweight | 0.016 | 0.073 | 0.009-0.617 |
| Obesity | 0.652 | 0.654 | 0.103-4.136 |

# 2、Supplementary Figure


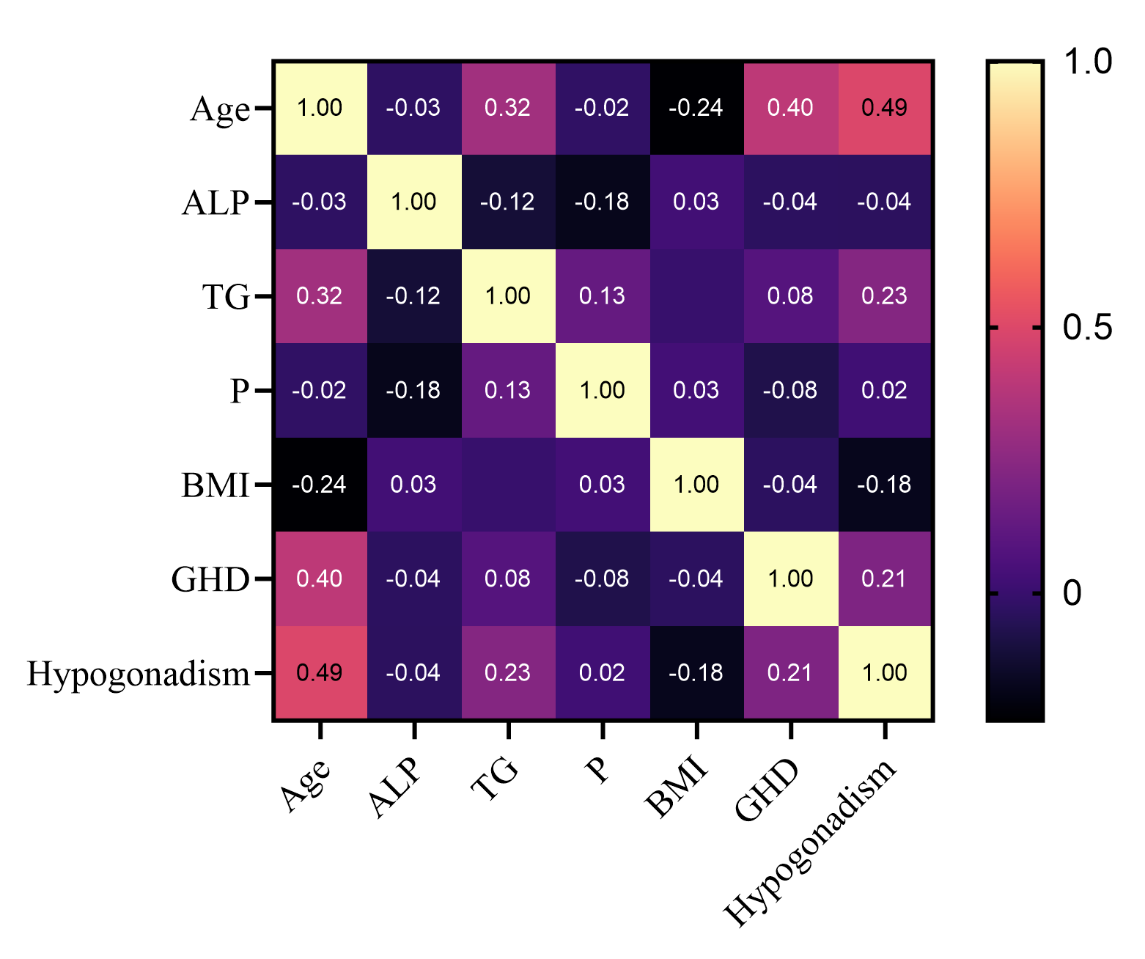


Figure1 Correlation Heatmap
